# Protection of Skin Fibroblasts from Infrared-A-Induced Photo-Damage Using Ginsenoside Rg3(S)-Incorporated Soybean Lecithin Liposomes

**DOI:** 10.4014/jmb.2210.10048

**Published:** 2022-12-29

**Authors:** Won Ho Jung, Jihyeon Song, Gayeon You, Jun Hyuk Lee, Sin Won Lee, Joong-Hoon Ahn, Hyejung Mok

**Affiliations:** Department of Bioscience and Biotechnology, Konkuk University, Seoul 05029, Republic of Korea

**Keywords:** Rg3(S), soybean lecithin liposome, fibroblast, infrared-A, photo-damage

## Abstract

Protection of skin cells from chronic infrared-A (IRA) irradiation is crucial for anti-photoaging of the skin. In this study, we investigated the protective activity of Rg3(S) and Rg3(S)-incorporated anionic soybean lecithin liposomes (Rg3/Lipo) with a size of approximately 150 nm against IRA-induced photodamage in human fibroblasts. The formulated Rg3/Lipo showed increased solubility in aqueous solution up to a concentration of 200 μg/ml, compared to free Rg3(S). In addition, Rg3/Lipo exhibited superior colloidal stability in aqueous solutions and biocompatibility for normal human dermal fibroblasts (NHDFs). After repeated IRA irradiation on NHDFs, elevated levels of cellular and mitochondrial reactive oxygen species (ROS) were greatly reduced by Rg3(S) and Rg3/Lipo. In addition, cells treated with Rg3/Lipo exhibited noticeably reduced apoptotic signals following IRA irradiation compared to untreated cells. Thus, considering aqueous solubility and cellular responses, Rg3/Lipo could serve as a promising infrared protector for healthy aging of skin cells.

## Introduction

Ginsenoside Rg3, one of the main components of steamed Korean ginseng (*Panax ginseng* Meyer), has been widely considered as a representative bioactive component for the treatment of diverse diseases such as metabolic syndromes and cancers owing to its anti-inflammatory, antioxidant, and anti-cancer effects [1, 2]. In particular, recent studies have shown that Rg3 has excellent protective effects on skin cells against UV-induced aging via gene regulation including the reduction of matrix metalloprotease-1 and interleukin-6 gene expression, as well as increased collagen gene expression in skin fibroblasts and keratinocytes [3, 4]. However, the biological benefits of Rg3 against photodamage by natural levels of infrared-A (IRA; wavelength of 700–1400 nm) irradiation without heat effects have not yet been studied in detail. Previously, we demonstrated that repeated IRA irradiation under physiological conditions significantly reduced cell proliferation and induced cellular apoptosis and senescence [5]. Therefore, it is necessary to evaluate the biological activity of Rg3 in the presence of IRA irradiation at natural levels in skin cells.

Despite the wide range of Rg3 antioxidant and anti-inflammation activity, its applications are still limited because of poor aqueous solubility and in vivo instability [2, 6, 7]. To improve the solubility and stability of Rg3, nanosized materials including nanoparticles, micelles, and liposomes, have been harnessed to encapsulate and stabilize Rg3 in aqueous solutions, which exhibited promising anti-cancer and anti-inflammatory effects in vivo [8-10]. However, there are very few studies on Rg3-incorporated nanomaterials, such as liposomes and solid lipid nanoparticles, for anti-photoaging in skin cells. Lipid-based formulations have been developed as biocompatible and feasible nanomedicines for the delivery of active molecules via the oral, topical, and inhalation routes. In particular, a natural combination of phospholipids, such as soybean lecithin (SL), which mainly comprises phosphatidylcholine, phosphatidylinositol, and phosphatidylethanolamine, has been widely investigated for lipid-based formulations owing to its eco-friendly processes, availability for large-scale production, and relatively low cost [11, 12]. Several SL-based formulations have been clinically approved by the United States Food and Drug Administration for transdermal delivery of bioactive molecules [12, 13]. Accordingly, SL-based liposomes have received attention in the cosmetics industry as practical carriers for the transdermal delivery of active molecules for anti-aging of skin.

To demonstrate the effects of Rg3 on IRA-induced photo-damage, normal human dermal fibroblasts (NHDFs) were repeatedly exposed to natural levels of IRA irradiation in the presence and absence of Rg3(S). To improve bioavailability in aqueous environments, Rg3(S) was incorporated into SL-based liposomes via conventional thin-film casting and hydration processes. Encapsulation efficiency, size, and morphology of Rg3(S)-incorporated SL liposomes (Rg3/Lipo) were examined by reverse-phase high-performance liquid chromatography (RP-HPLC), dynamic light scattering (DLS), and transmission electron microscopy (TEM), respectively. Finally, we demonstrated Rg3/Lipo as a novel cosmetic biomaterial in terms of its colloidal stability, biocompatibility, and protective effects against IRA. The colloidal stability of Rg3/Lipo in distilled water at 4°C and -18°C was investigated after up to 14 days using DLS. After six rounds of IRA irradiation over 10 days in the presence of the formulated Rg3/Lipo, the levels of intracellular reactive oxygen species (ROS) and apoptotic signals were examined qualitatively and quantitatively.

## Materials and Methods

### Materials

Ginsenoside Rg3 20(S) (> 98% purity) was purchased from Chengdu Biopurify Phytochemicals, Ltd. (China). L-α-lecithin (soybean), chloroform, dimethyl sulfoxide (DMSO), fluorescein isothiocyanate (FITC), 2′,7′-dichlorofluorescin diacetate (DCFDA), trypan blue, 37% formaldehyde, mounting medium (Fluoroshield with and without 4′,6-diamidino-2-phenylindole), Bright-Line hemacytometer, and phosphate-buffered saline (PBS) were purchased from Sigma-Aldrich (USA). Fetal bovine serum (FBS), Dulbecco’s modified Eagle medium (DMEM), trypsin-EDTA, and penicillin/streptomycin were obtained from Gibco (USA). Cell Counting Kit-8 (CCK-8) was obtained from Dojindo Laboratories (Japan). The FITC Annexin V apoptosis detection kit I was obtained from BD Biosciences (USA). The MitoSOX Red mitochondrial ROS indicator was purchased from Thermo Fisher Scientific (USA).

### Preparation of Rg3/Lipo

The liposomes were formulated using conventional thin-film casting and hydration processes. Lecithin (13.7 mg) in chloroform and Rg3(S) (1.37 mg) in DMSO were mixed in a 10:1 weight ratio and evaporated in a rotary evaporator at 50°C. The resulting thin film was hydrated with an ammonium phosphate buffer (300 mM, pH 7.4) and sonicated for 5 min using a tip sonicator (Branson Digital Sonifier 450; USA) at 30% amplitude, 59 s on/12 s off. After sonication, the resulting solutions were centrifuged at 10,000 rpm for 10 min to remove free Rg3(S), and the supernatant was purified via dialysis using distilled water at room temperature for 2 days. After filtering the liposomes through a 0.22 μm filter (Merck Millipore, Ireland), the hydrodynamic size was measured by DLS at 25°C using a Nano-S device (Malvern Instrument Ltd, UK). All data are presented as the mean ± standard deviation.

### Characterization of Rg3/Lipo

For TEM analysis, Rg3/Lipos (0.3 μg) were placed on a 300-mesh copper grid and air-dried. After washing the grid three times with distilled water, the samples were stained with 2% uranyl acetate solution. Excess uranyl acetate was removed by washing and the grids were observed using a Bio-TEM instrument (Tecnai G 2 Spirit Twin; FEO, USA). For aqueous stability assay, Rg3/Lipos (10 μg) in PBS solution were stored at 4°C and -18°C for predetermined time intervals (1, 4, 7, 10, and 14 days). The sizes of the incubated Rg3/Lipos were measured using DLS.

To determine the encapsulation efficiency, Rg3/Lipos were purified by dialysis to remove free Rg3(S) and the solvent. After freeze-drying, the resulting powder was dissolved in 500 μl methanol and the amount of Rg3(S) was analyzed using RP-HPLC (Agilent Technologies, USA) equipped with a diode-array detector (Agilent Technologies) on a Varian polar C18 reverse-phase column (4.60 × 250 mm, 0.45 μm; Varian, USA) to calculate Rg3(S) and lecithin [7]. The mobile phase consisted of 0.1% formic acid in water and acetonitrile [14]. The flow rate was set at 1 ml/min. The eluent was analyzed at a wavelength of 205 nm. The encapsulation efficiency was calculated as follows: Rg3(S) encapsulation efficiency (%) = encapsulated amount of Rg3(S)/initial amount of Rg3(S) × 100.

### Cell Viability Assay

NHDFs were maintained in DMEM supplemented with 10% FBS, 100 U/ml penicillin, and 100 μg/ml streptomycin at 37°C in a humidified atmosphere containing 5% carbon dioxide. NHDFs were seeded in 96-well plates at a density of 3 × 10^3^ cells/well and incubated at 37°C for 24 h before treatment. After incubation of the cells with liposomes, Rg3(S), and Rg3/Lipo (0, 0.1, 0.3, 0.5, 1, 2, 4, 8, and 15 μg/ml) samples for 24 h at 37°C, the cell viability was examined using the CCK-8 assay, according to the manufacturer’s protocol.

### ROS Assay

NHDFs were plated on 35-mm dishes at a density of 3 × 10^4^ cells/dish. After 24 h of incubation, the cells were incubated with Rg3(S) and Rg3/Lipo at an Rg3 concentration of 4 μg/ml. IRA irradiation was performed on the transparent lids of the cell culture dishes using a customized solar IRA generator at an irradiance of 38-42 mW/cm^2^ for 4 h per day (once a day), in accordance with our previous study [15]. During IRA irradiation, cell culture dishes were placed on a water-circulating plate equipped with a circulating water bath (Lab Korea, Korea) at a temperature of 34°C. Cells in the culture media were also irradiated as controls (Con). After repeated IRA exposure (three rounds) over 3 days, cells were harvested and stained with trypan blue (0.4%) in PBS solution for cell counting on the hemacytometer using the microscope. The counted cells were seeded at a density of 2 × 10^4^ cells in new 18-mm dishes coated with polylysine. After a further three rounds IRA exposures (total number of irradiations: six rounds over 10 days), cells were washed twice with PBS solution and stained with DCFDA (20 μM) in serum-free DMEM without phenol red and incubated for 30 min at 37°C in the dark. After washing the cells twice with PBS, the medium was replaced with fresh serum-free DMEM without phenol red and incubated for 20 min at 37°C in the dark. Fluorescence images of DCFDA within the cells were visualized using an inverted fluorescence microscope (Nikon Eclipse Ti2, Japan).

Mitochondrial ROS levels were determined using the MitoSOX Red mitochondrial superoxide indicator, according to the manufacturer's protocol. Briefly, after the last irradiation, the cells were washed twice with PBS solution and treated with MitoSOX (5 μM) solution for 10 min. After incubation, the cells were washed with PBS solution, covered with mounting solutions, and visualized by inverted fluorescence microscope. The fluorescence intensity within the cells was analyzed using the ImageJ software (National Institutes of Health, USA; http://rsb.info.nih.gov/ij/).

### Annexin V Staining

NHDFs were plated on 35-mm dishes at a density of 3 × 10^4^ cells/dish. After 24 h of incubation, the cells were incubated with Rg3(S) and Rg3/Lipo at an Rg3(S) concentration of 4 μg/ml. The cells were then irradiated with IRA for six rounds at 34°C as described previously. After the last irradiation, the cells were washed with PBS and binding buffer (10 mM HEPES (pH 7.4), 140 mM NaCl, and 2.5 mM CaCl_2_). The cells were then stained with FITC-labeled annexin V in a binding buffer and incubated for 15 min at room temperature in the dark. Non-irradiated cells (Cell) were also stained with FITC-labeled annexin V. After washing with the binding buffer, the cells were incubated three times for 1 min with PBS solution containing 5% FBS. After washing the cells with PBS solution, they were fixed with formaldehyde (3.7%) in PBS solution for 10 min and covered with a mounting medium. Fluorescence within the cells was visualized using inverted fluorescence microscope and quantified using ImageJ software.

### Statistical Analysis

Data in this study represent the mean values of independent measurements. Error bars indicate the standard deviations of each experiment. Statistical analysis was performed using Student’s t-test. Statistical significance was assigned for *p* < 0.05 (95% confidence level).

## Results and Discussion

### Characterization of Rg3/Lipo

**Fig. 1:**
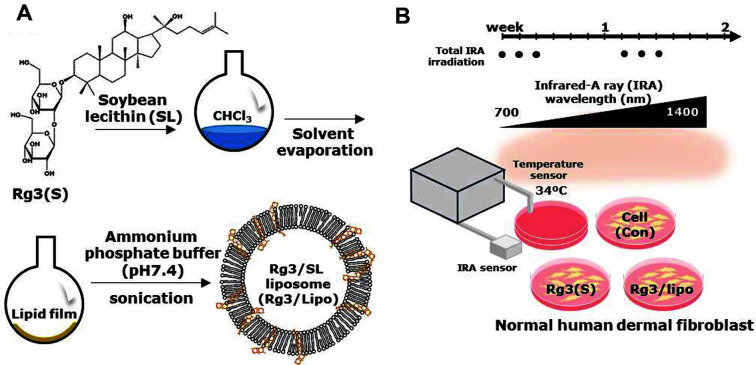
(A) Schematic illustration for the preparation of Rg3/Lipo, and (B) six rounds of IRA irradiation on normal human dermal fibroblasts (NHDFs) in the presence of Rg3/Lipo at 34°C.

Fig. 1A shows the formulation processes for the preparation of Rg3/Lipo using ginsenoside Rg3(S) and SL via thin-film casting and hydration processes. Previous studies have reported that the stereoselectivity of the hydroxyl group on C-20 of ginsenoside Rg3 resulted in two types of Rg3 stereoisomers, Rg3(S) and Rg3(R), which are crucial for biological activity [16-18]. In particular, Rg3(S) showed a more promising reduction in ROS by UV radiation in human keratinocytes and fibroblasts than Rg3(R) [17]. In addition, natural SL, which is mainly composed of phosphatidylcholine, has been popularly used for cosmetics for a long time due to its biocompatibility and deep tissue penetration [19, 20]. Accordingly, ginsenoside Rg3(S) and SL were used to prepare Rg3/Lipo in this study. After the evaporation of CHCl_3_ and the hydration of the thin film with ammonium phosphate buffer via sonication, nanosized Rg3/Lipos were successfully prepared. Owing to the amphiphilic characteristics of the ginsenoside Rg3 20(S) isomer, the hydrophilic sugar moiety of amphiphilic Rg3(S) could be located on the exterior or interior of liposomes, while the hydrophobic moiety could be embedded into the lipid layer after formulation. To examine the bioactivity of Rg3/Lipo for protection against IRA-induced photodamage, NHDFs were selected as a model fibroblast because of their vulnerability to IRA irradiation, as described in our previous study [5]. To mimic natural IRA conditions, the natural level of IRA (38-42 mW/cm^2^) was irradiated for 4 h per day over 10 days [15]. To protect cells from heat stress generated by IRA irradiation, the temperature of the medium was controlled at 34°C via a cooling circulator. After six rounds of irradiation, the levels of cell proliferation, intracellular ROS, and apoptotic signals were assessed quantitatively and qualitatively.

**Fig. 2:**
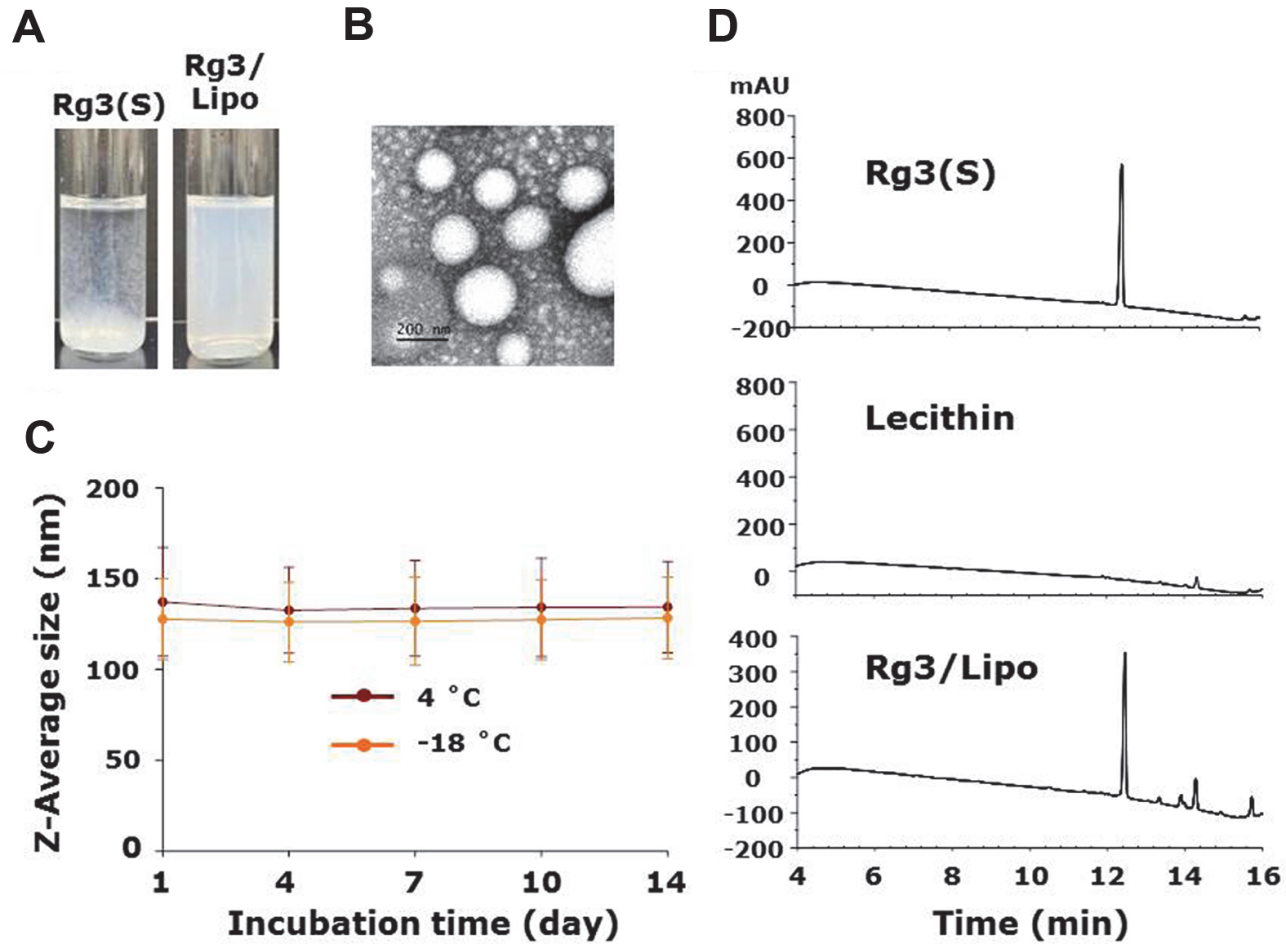
(A) Photographic images of Rg3(S) and Rg3/Lipo in aqueous solution, (B) transmission electron microscopy (TEM) images of Rg3/Lipo, (C) the hydrodynamic sizes of Rg3/Lipo after 14 days at 4°C and - 18°C, and (D) high performance liquid chromatography (HPLC) analysis of Rg3(S), lecithin, and Rg3/Lipo.

Fig. 2A shows images of Rg3(S) and Rg3/Lipo in ammonium phosphate buffer (pH 7.4). Owing to the poor aqueous solubility of Rg3(S) (< 0.02 mg/ml), severe aggregation was observed in free Rg3(S) [21]. However, Rg3/Lipo samples were homogeneously dispersed up to 0.20 mg/ml in aqueous solution without any noticeable aggregates, which indicates a ten-fold increase in the solubility of Rg3(S). The hydrodynamic sizes of Lipo and Rg3/Lipo samples in water were measured using DLS. The diameters of Lipo and Rg3/Lipo samples were 131.7 ± 6.1 and 136.4 ± 16.9 nm, respectively. The surface charge of Lipo and Rg3/Lipo samples were -13.8 ± 0.1 and -4.8 ± 2.3 mV, respectively. In previous studies, formulations of drugs and bioactive molecules within both cationic and anionic liposomes have shown greatly enhanced skin penetration by several times compared to naked molecules via different mechanisms [22, 23]. In particular, anionic liposomes below 150 nm penetrated through skin tissues easily and delivered their cargos into deep skin layers [13, 24, 25]. In this study, we successfully prepared anionic SL liposomes with sizes below 200 nm, which might allow for the preferred skin penetration and delivery of Rg3(S). The morphology of Rg3/Lipo was observed by TEM. As shown in Fig. 2B, spherical liposomes were observed in TEM images. The colloidal stability of Rg3/Lipo was assessed by DLS after incubation for 14 days at 4°C and -18°C, respectively (Fig. 2C). There were negligible changes in particle size after 14 days at 4°C and -18°C. The diameter of Rg3/Lipo samples after incubation for 7 days and 14 days at 4°C was 133.6 ± 26.3 and 134.4 ± 25.1 nm, respectively. These data indicate the superior stability of our Rg3/Lipos in aqueous solutions.

To determine encapsulation efficiency, the amount of Rg3(S) within the liposomes was quantitatively analyzed by HPLC. As shown in Fig. 2D, Rg3(S) and Rg3/Lipo samples were detected at a retention time of 12.5 min while a negligible signal was observed in lecithin. In this study, encapsulation efficiency of Rg3(S) within SL liposomes was 75.2 ± 11.5%. In a previous study, encapsulation efficiency of Rg3 within liposomes composed of egg yolk derived phosphatidylcholine and cholesterol was approximately 80%, which is consistent with our study [6]. In addition, considering the encapsulation efficiency of hydrophilic molecules was approximately 50% in a previous study, the high encapsulation efficiency of Rg3 within SL liposomes might be attributed to amphiphilic properties of Rg3 [24].

### Cell Viability Assay

**Fig. 3:**
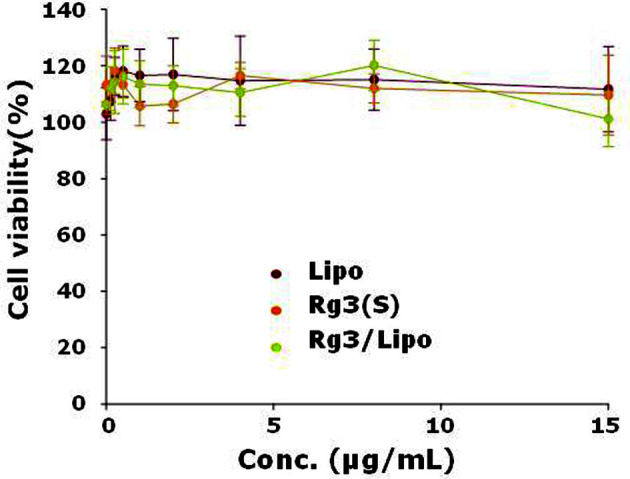
Cell viability of Rg3(S) and Rg3/Lipo for NHDFs at different concentrations.

After treatment of NHDFs with Rg3(S) or Rg3/Lipo for 24 h, cell viability was assessed using the CCK-8 assay. Owing to the poor solubility of Rg3(S), Rg3(S) concentration in the cell viability assay was below 20 μg/ml. As shown in Fig. 3, both Rg3(S) and Rg3/Lipo exhibited excellent biocompatibility up to an Rg3(S) concentration of 15 μg/ml. The cell viabilities of Rg3(S), vacant liposomes, and Rg3/Lipo at an Rg3(S) concentration of 15 μg/ml were 109.6 ± 14.1, 111.7 ± 15.2, and 101.1 ± 9.8%, respectively.

### ROS Assay

**Fig. 4:**
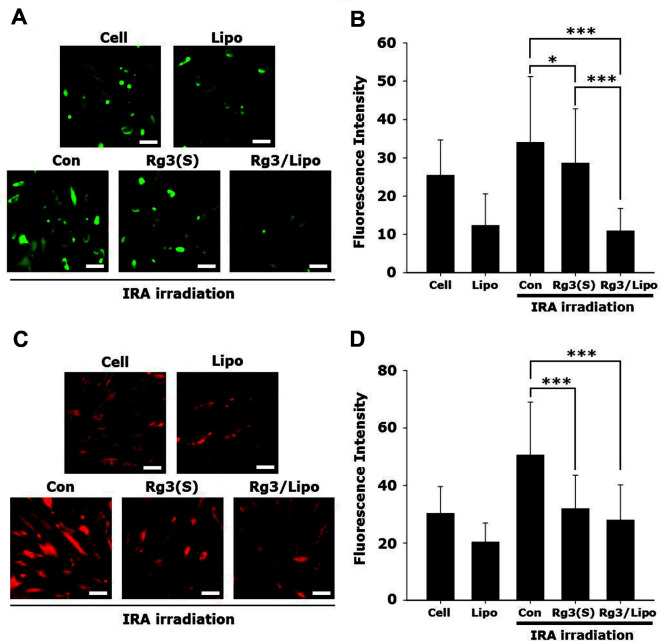
(A) Fluorescence microscopic images of cells, (B) relative green fluorescence intensity within cells after 2′,7′-dichlorofluorescin diacetate (DCFDA) assay, (C) fluorescence microscopic images of cells, and (D) relative red fluorescence intensity within cells after MitoSOX assay. *p < 0.05; ***p < 0.001.

To examine whether Rg3(S) could reduce cellular and mitochondrial ROS generated by IRA irradiation, NHDFs were irradiated as described above. The antioxidant activities of Rg3(S) and Rg3/Lipo were comparatively investigated by DCFDA and MitoSOX assays at an Rg3(S) concentration of 4 μg/ml. After the last IRA irradiation, the level of cellular ROS was determined using the DCFDA assay, as shown in Fig. 4A. Both the untreated cells (Cell) and those treated with liposomes (Lipo) exhibited negligible green fluorescent signals. However, the IRA-treated cells (Con) showed strong green fluorescence, indicating successful generation of intracellular ROS by IRA irradiation. As expected, the level of the green fluorescence signal was greatly reduced by Rg3(S) and Rg3/Lipo due to a considerable decrease in intracellular ROS. The level of green fluorescence intensity was quantitatively analyzed using the ImageJ software, as shown in Fig. 4B. The cellular ROS level induced by IRA irradiation was significantly reduced by Rg3/Lipo compared to Rg3(S). The relative intensities of green fluorescence in Con, Rg3(S), and Rg3/Lipo were 34.0 ± 17.2, 28.6 ± 14.2, and 10.9 ± 5.9, respectively.

To examine the effects of IRA irradiation on mitochondrial ROS, cells were stained with MitoSOX after IRA irradiation (Fig. 4C). A strong red fluorescent signal was observed in Con because of high levels of mitochondrial ROS, whereas noticeably reduced fluorescence intensity was observed in Rg3(S) and Rg3/Lipo. The relative intensities of red fluorescence in Con, Rg3(S), and Rg3/Lipo were 50.6 ± 18.4, 31.9 ± 11.6, and 27.9 ± 12.3, respectively (Fig. 4D). This result indicated that the level of mitochondrial ROS was successfully reduced by Rg3(S) and Rg3/Lipo, which was similar to that in Cell without IRA irradiation. In previous studies, the antioxidant activity of Rg3 restored the mitochondrial membrane potential in UV-damaged fibroblasts [3, 4]. In this study, we demonstrated that Rg3(S) and Rg3/Lipo show a promising decrease in mitochondrial ROS induced by IRA irradiation.

According to our previous study, IRA radiation with a wavelength of 700–1400 nm plays a crucial role in skin damage and aging, even without heat stress [5]. A previous study reported that the expression levels of genes related to skin aging and inflammation, such as interleukin-6 and matrix metalloproteinase-1, could be regulated by Rg3 after IRA irradiation without heat control [4]. However, the effects of Rg3 on cellular and mitochondrial ROS levels, cellular apoptosis, and cell aging in the presence of IRA irradiation without heat stress have not yet been examined. As shown in Fig. 4, we demonstrated that Rg3(S) reduced both cellular and mitochondrial ROS generated by IRA at physiological temperatures. Considering that skin aging could be triggered by intracellular ROS, these data suggest that Rg3(S) could be harnessed as a promising bioactive molecule for IRA protection in cosmetics. In addition, it should be noted that Rg3/Lipo showed a superior reduction of cytoplasmic ROS levels compared to free Rg3(S). In previous studies, exterior Rg3 on liposomes allowed for the targeted delivery of liposomes to brain capillary endothelial cells through interactions of Rg3 with glucose transporter 1 and subsequent receptor-mediated endocytosis [2, 26]. Considering glucose transporter 1 expression in human fibroblasts, exterior Rg3(S) on Rg3/Lipo might be likely to facilitate intracellular delivery of Rg3/Lipo to NHDFs and greatly reduce intracellular ROS [27].

### Annexin V Staining

**Fig. 5:**
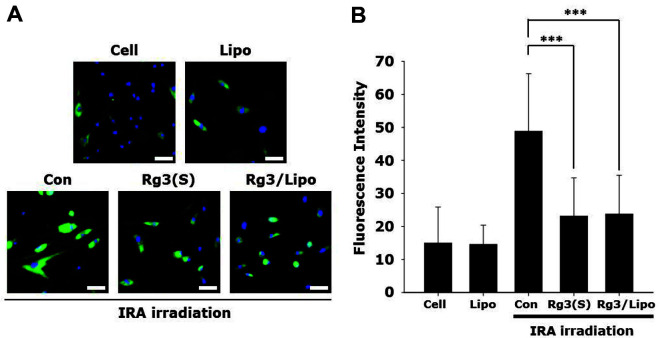
(A) Fluorescence microscopic images of cells, and (B) relative fluorescence intensity within cells after annexin V staining. ***p < 0.001.

Quantitative analysis of exterior phosphatidylserine in the plasma membrane has been considered a convincing marker for cellular apoptosis [28]. To examine the extent of apoptosis induced by IRA irradiation, NHDFs were stained with fluorescence-labeled annexin V after six rounds of IRA irradiation. Cell and Lipo samples showed negligible fluorescence intensity of annexin V, while the green signal of annexin V was significantly increased in Con by IRA irradiation (Fig. 5A). However, annexin V intensity was noticeably reduced by Rg3(S) and Rg3/Lipo at an Rg3(S) concentration of 4 μg/ml. The relative fluorescence intensity was quantitatively analyzed, as shown in Fig. 5B. The annexin V intensity in Cell, Lipo, Con, Rg3(S), and Rg3/Lipo was 7.3 ± 3.7, 7.6 ± 4.8, 16.5 ± 8.8, 3.5 ± 2.3, and 4.7 ± 2.3, respectively. According to a previous study, the UV-induced pro-apoptotic factor cytochrome-c was reduced by Rg3(S), which might be one of the potential mechanisms of decreased annexin V levels by Rg3/Lipo [3, 4].

In this study, we first demonstrated protective effects of Rg3(S) against natural level of IRA irradiation at physiological temperature in skin cells. In addition, we successfully formulated anionic Rg3/Lipo with a size of approximately 150 nm. Rg3/Lipo greatly increased the solubility of Rg3(S) in aqueous solutions up to a concentration of 200 μg/ml and exhibited excellent aqueous stability for two weeks. After repeated IRA irradiation, Rg3/Lipo exhibited noticeably reduced cellular and mitochondrial ROS and apoptotic signals. Thus, Rg3/Lipo could serve as a promising infrared protector for healthy aging of skin cell
